# Total functioning capacity scale in Huntington's disease: natural course over time

**DOI:** 10.1007/s00415-024-12771-w

**Published:** 2025-01-15

**Authors:** K. F. van der Zwaan, S. Feleus, O. M. Dekkers, R. A. C. Roos, S. T. de Bot

**Affiliations:** 1 LUMC Department of Neurology, Albinusdreef 2, 2333 ZA Leiden, The Netherlands; 2 LUMC Department of Clinical Epidemiology, Albinusdreef 2, 2333 ZA Leiden, The Netherlands

**Keywords:** Huntington's disease, Functional status, Disease progression, Movement disorders

## Abstract

**Background and objectives:**

The total functioning capacity (TFC) assessment has been integral to Huntington's disease (HD) research and clinical trials, measuring disease stage and progression. This study investigates the natural progression of function in HD, focusing on changes in TFC scores related to age and CAG-repeat length, and evaluates TFC's strengths and weaknesses in longitudinal studies.

**Methods:**

Using Enroll-HD platform's clinical dataset version 5, including Registry-3, we analysed data from 21,079 participants, with 16,083 having an expanded CAG repeat. Our final analysis encompassed 15,527 patients and 52,457 visits, with TFC scores ranging from 0 to 13.

**Results:**

Alluvial charts show that most individuals maintain maximum functional capacity over time. 3505 individuals experienced change in TFC scores, over the subsequent 4 years, 2224 (64.1%) experienced declining TFC scores, while 661 (18.6%) showed improvement within a year. The remaining 17.3% exhibited stable TFC scores. Age-related changes followed a specific sequence: occupation, household chores/finances, daily living, and care. Longer CAG-repeat lengths were linked to earlier functional decline, with some geographic regions showing earlier losses in specific domains. Reduced penetrance CAG-repeat groups exhibited different trajectories from full penetrance HD participants.

**Discussion:**

When we focus on those who experienced a change in TFC score, the number of HD patients with regained functional capacity is substantial, even considering interrater variability, which may influence outcome assessments in clinical trials. The TFC effectively reflects changes in functional domains as intended. Analysis of the reduced penetrance group suggests potential selection biases in seeking medical attention earlier and for reasons unrelated to HD.

## Introduction

Disease progression in neurodegenerative disorders can be regarded as a description of the natural course of the disease; a process that leads to an irreversible decline in functioning over time.

Currently, much research focuses on identifying and optimising clinical outcome measures to quantify disease progression. Moreover, although clinical outcome measures in movement disorders like Parkinson’s disease (PD) and Huntington’s disease (HD) traditionally emphasize individual signs such as motor disturbances (e.g., bradykinesia in PD and chorea in HD), cognitive signs, and psychiatric behaviours, these Clinical Outcome Assessments (COAs) are primarily effective in assessing specific signs rather than providing a comprehensive measure of disease description and progression. Although bradykinesia in PD and chorea in HD are pivotal symptoms of the diseases, these neurodegenerative disorders often lead to or even start with psychiatric disturbances and cognitive impairments [1, 2]. Hence, because of the diverse symptomatology, and especially, since HD manifests heterogeneous disease expression, a single sign cannot be used as a suitable clinical outcome measure for overall disease description and progression.

In HD, the presence of the mutant cytosine-adenosine-guanine repeat length (CAG-repeat length) can be determined many years before disease onset [3]. While predictive genetic testing for some other neurodegenerative conditions, such as *C9ORF72*-related frontotemporal dementia (FTD), has also become possible, the possibility of early identification remains a rather distinctive aspect of HD [4]. Through predictive testing, it is possible to track expanded gene carriers of the disease years before it becomes clinically manifest. Shoulson and Fahn (1979) determined the clinical care needs of one hundred-and-twenty-four HD patients [5]. As a result, they proposed a multi-faceted designation of functional capacity in HD. This classification system was later assimilated into the Unified Huntington's Disease Rating Scale as the Total Functioning Capacity Scale (TFC) [6]. Despite recent advances in HD research introducing alternative measures of disease progression, such as the composite Unified Huntington Disease Rating Scale (cUHDRS), which incorporates various clinical measures including age, CAG-repeat length, cognitive assessments like the Symbol Digit Modalities test, Total Motor Score, and TFC, a recent review and recognition by the Food and Drug Administration (FDA) reaffirmed the essential role of TFC as an assessment tool and an important outcome measure in Huntington's Disease [7, 8]. Moreover, ten years ago, the COHORT study revealed longitudinal changes in clinical features of HD, indicating a mean of 0.6 point (95% CI 0.5–0.7) annual decline in function [9]. In addition, an important study on Total Functional Capacity (TFC) assessment demonstrated its rate of decline across different disease stages, allowing for sample size calculations in the development of therapeutic agents aimed at slowing decline [10]. Consequently, the TFC assessment is now widely used in clinical trials as a primary endpoint and is assumed to be one of the most robust measures of disease progression [8].

The TFC is also used to determine the disease stage in HD. Recent progress has also been made here. For instance, the HD-Integrated Staging System (HD-ISS) categorizes progression into four stages using markers like CAG length, striatum atrophy on MRI, total motor score, symbol digits modalities test and functional decline. Clinical trials are increasingly adopting HD-ISS stages as criteria for participant inclusion and exclusion. This approach enables the inclusion of individuals with only mild atrophic pathology in clinical trials, potentially facilitating disease modification at an early stage [11]. Given that the HD-ISS incorporates the TFC, understanding how the TFC evolves during the natural course of the disease is essential. Observations like these can provide valuable context for the effectiveness of early interventions and enhance the overall assessment of disease progression. Moreover, despite this advancement, many trials still use the classification of disease stages depending on TFC scores to establish in- and exclusion criteria, or the TFC is once again included in a new staging system.

While novel Huntington's Disease staging tools, such as the HD-ISS, and measures for disease progression are emerging in HD research, the Total Functional Capacity remains highly respected and widely used. It is likely that it will continue to be a mainstay of future studies, especially as long as the FDA recognizes it as a crucial outcome measure in clinical HD trials. Additionally, its informative nature in clinical settings further solidifies its importance, especially, as lower TFC scores are related to higher disease burden [9]. Surprisingly, there has been a notable gap in research on the natural course of the TFC over the years in HD, particularly in terms of exploring the diversity and direction of changes and delving into the details of changes within specific TFC domains.

Therefore, our study aims to thoroughly investigate the natural course of Total Functional Capacity as assessed within cohort studies. Furthermore, we seek to provide observational insights into the age at which changes occur in different TFC domains, taking into account the individual's CAG-repeat length. This fundamental exploration of TFC in the context of natural disease progression will enhance our understanding of the evolving functional dynamics in HD over time.

## Methods

This study combined the Enroll-HD and Registry3 datasets provided in the Enroll-HD's fifth periodic dataset, released on October 31st, 2020. Registry3 was a European study that started in 2004 and ended in 2015. It continued as part of Enroll-HD (starting in 2012), which is ongoing. Enroll-HD (NCT01574053) is a global clinical research platform designed to facilitate clinical research in Huntington's Disease and made available by CHDI Foundation, Inc. Core datasets are collected annually from all research participants as part of this multi-centre cohort study. Data are monitored for quality and accuracy using a risk-based monitoring approach. In addition, all sites obtained and maintained local ethical approval [12].

The combined datasets yielded information on 21,079 participants, 16,083 of whom have an expanded CAG-repeat (CAG ≥ 36) and 4996 controls (CAG < 36). The current study focuses on individuals with an expanded CAG repeat between 36 and 51 with a visit with a TFC score, which results in a dataset of 15,527 participants. However, a distinction is made between those with the reduced penetrant form of HD (RP: CAG ≥ 36 and < 40) and the fully penetrant form of HD (FP: CAG ≥ 40). The age at onset of HD is negatively correlated to the CAG-repeat length [13, 14]. Hence, if individuals with RP are affected by HD, this is often at an older age than those with FP [14].

We used the Unified Huntington's Disease Rating Scale—Total Functioning Capacity, which provides a sum score ranging from 0 to 13. Lower scores indicate reduced functional capacities. It consists of five functional subdomains, respectively: occupation (range 0–3), finances (range 0–3), household chores (range 0–2), activities of daily living (range 0–3), and care level (range 0–2), for the total scale see Appendix Table 4 [5, 6].

### Objectives

The first objective is to investigate the natural course and potential fluctuation of the TFC score over time, using data from the ENROLL-HD and Registry studies. To achieve this, we will visualize annual TFC score changes through alluvial plots, which will represent the functional changes experienced by participants across study visits. In particular, this analysis will focus on the first 4 years following the onset of functional decline to assess the trajectory of changes in greater detail.

The second objective is to examine the sequence of functional changes across the five TFC domains: occupation, finances, household chores, activities of daily living, and care level. We will analyse changes by disease stage of functional decline. Additionally, we will explore correlations between these functional changes and the CAG-repeat length. Here, the endpoints will include determining the percentage and sequence of TFC domain changes across different disease stages, as well as assessing whether equal points lost on the TFC represent equivalent functional loss across different domains. We explore the difference between premanifest and manifest participants. To better understand the variability in functional decline, we will also analyse the median age of decline within each TFC domain, stratified by CAG-repeat length groups.

The third objective focuses on exploring the relationship between the decline in TFC scores and factors such as CAG repeat length, age, predicted age at onset, sex, education level, and place of residence. To address this objective, we will evaluate the general associations between these variables and the observed changes in functional capacity. The endpoints will involve statistical analyses using univariate linear models, enabling to explore how demographic and genetic factors influence the timing and nature of TFC decline in patients with HD.

Finally, the study's fourth, more explorative, objective is to distinguish potential differences between individuals with fully penetrant (FP) and reduced penetrant (RP) forms of HD. By comparing the TFC score trajectories between these two groups, the endpoint will focus on identifying any significant variations in patterns of functional decline, which may provide valuable insights into the differential progression of HD based on penetrance.

### Statistical analyses

R 4.1.2 [15], the tidyverse 1.3.1[16], and ggalluvial 0.12.3 [17] packages were used for statistical analyses. First, we constructed alluvial plots, representing changes in TFC scores per participant over annual study visits and in the 4 years after initial functional change onset. Since the TFC is an obligatory core assessment of Enroll-HD, missing data is already minimised in the dataset, and only complete case analyses are performed.

Second, we reported the distribution of changes in TFC scores per disease stage, as defined by Shoulson and Fahn [18], to show the percentage of change per TFC domain. The TFC score determines disease stages: viz. Stage I (TFC 11–13), Stage II (TFC 7–10), Stage III (TFC 4–6), Stage IV (TFC 1–3), and Stage V (TFC 0). We report the whole group and explore differences between premanifest and manifest participants.

Third, univariate linear models were used to determine whether sex, age, predicted age at onset (as determined by Langbehn et al.) [14], level of education (determined by the International Standard Classification of Education (ISCED)) or place of residence might be covariates of age at functional change. We did this for FP, and to explore the fourth endpoint compared FP to RP trajectories.

N.B. In our analysis, we explored multivariate linear regression. However, multicollinearity, particularly in the interaction between the region variable and the outcome variable for change (VIF > 10), undermined the statistical significance of our dependent variable.

### Data availability

Any researcher employed by a recognized research organization can open an Enroll-HD access account to obtain data and biosamples from Enroll-HD, subject to the researcher's employer/institution signing the appropriate data use agreement. More detailed access information is available at https://enroll-hd.org/for-researchers/access-data/.

## Results

### Total functional capacity scores

**Fig. 1:**
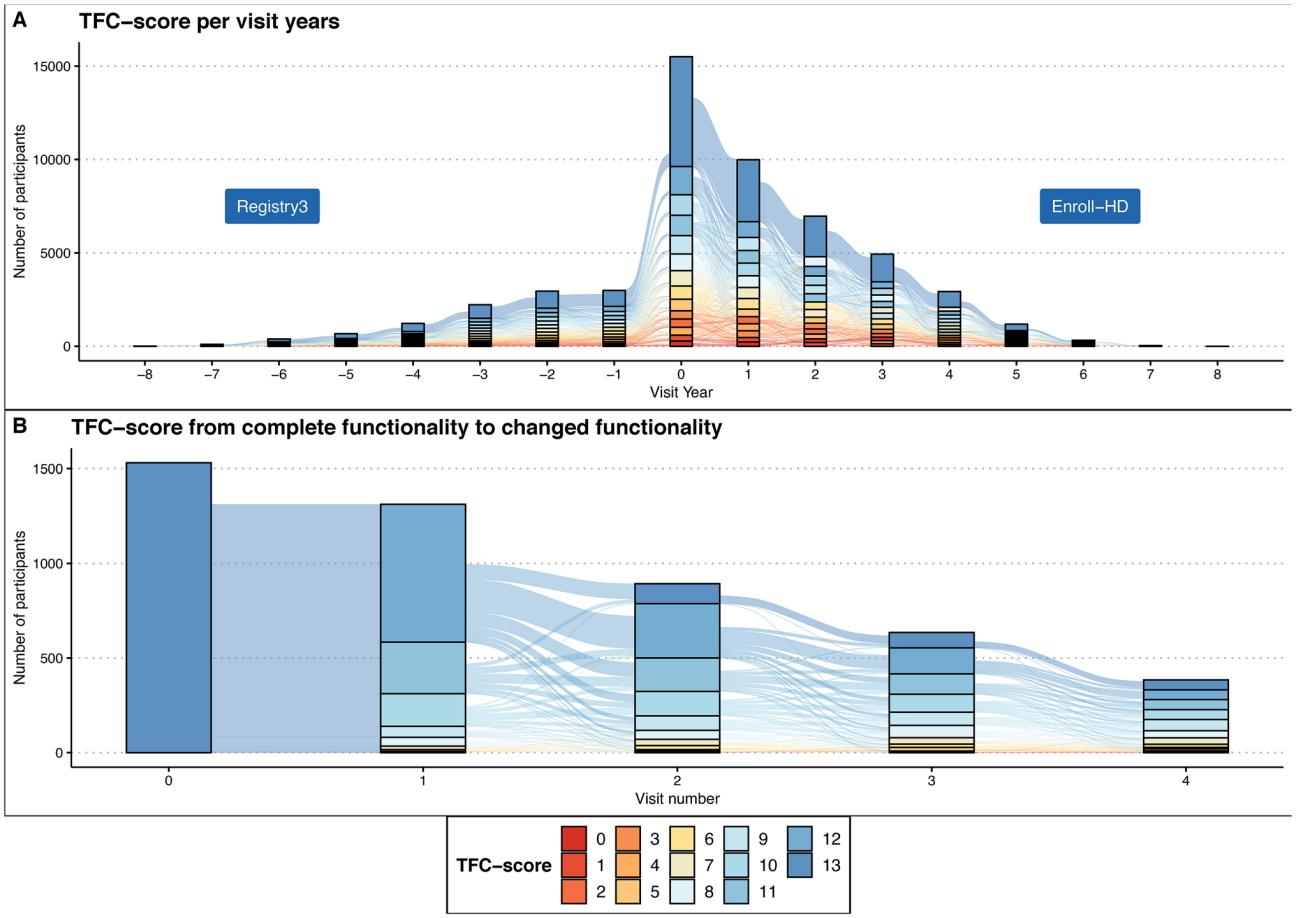
Alluvial graphs representing the natural course of TFC scores during Registry3 (left) and Enroll-HD (right) (**A**) and the course of TFC scores when a change in functionality is reported (**B**). In Fig (A**)**, visit year 0 = baseline Enroll-HD. All successive visits are part of the Enroll-HD study. All visits before 'visit year = 0' are part of the Registry3 study

The dataset for analyses consisted of 15,527 patients and 52,457 visits containing TFC scores, for demographical information refer to Table 1. Most participants in Registry3 and Enroll-HD had a maximum score of 13 on the TFC and remained stable over the years (Fig. 1A).

**Table 1:** The demographic of the cohort

	*N*	Percentage
Registry3 and Enroll-HD	21.079	–
CAG ≥ 36	16.083	–
CAG 36–51 and TFC scores	15.527	–
Sex		
Female	8419	54.2%
Male	7108	45.8%
Age (min/max)	18/94	–
CAG-groups		
Reduced penetrance 36–39	875	5.6%
40–43	8937	57.6%
44–47	4539	29.2%
48–51	1176	7.6%
Education		
ISCED 0	42	0.3%
ISCED 1	565	3.5%
ISCED 2	2522	15.5%
ISCED 3	4999	30.8%
ISCED 4	3036	18.7%
ISCED 5	4502	27.8%
ISCED 6	457	2.8%
Missing	101	0.6%
Place of residence		
Australasia	590	3.8%
Europe	10,262	66.0%
Latin America	156	1.0%
Northern America	4539	29.2%
Unique subjects TFC 13 and decline	1.531	–
Visit 1 (one year after TFC 13)	1.313	–
Visit 2 (two years after TFC 13)	893	–
Visit 3 (three years after TFC 13)	636	–
Visit 4 (4 years after TFC 13)	385	–

*ISCED 0* Early childhood educational development; preprimary education, *ISCED 1* Primary education or the first stage of basic education, *ISCED 2* Lower secondary education or second stage of basic education, *ISCED 3* Upper secondary education, *ISCED 4* Specific occupational training certificate, *ISCED 5* First stages of tertiary education, *ISCED 6* Degrees leading to advanced research qualifications

The cumulative amount of education level is higher than the unique subjects in the dataset used for analysis, this is because some subjects got higher education over time.

In the participants with a TFC of 13 who experienced a change in TFC-score in the first year (*n* = 2021), 55.3% changed from 13 to 12, 20.6% decreased from 13 to 11, and 13.2% decreased to 10 (Fig. 1B). With every larger step in TFC-score change, the group size decreased (i.e., a step-down from 13 to 2 being extremely rare). The year after the decline, 11.2% again scored the maximum of 13. Most of these individuals had scored 12 the year before, but not all.

By examining all consecutive visits after the initial change, it is possible to obtain a count of the overall change. Our analysis reveals that over 4 years after the initial change, 64.1% of individuals exhibited a further decline in TFC scores and 17.7% showed a stable TFC score during subsequent visits. In 18.6% of cases, an increased TFC score was seen at the subsequent visit. Of these, 7.6% had an improvement score of more than 1. An overview of the distribution of positive and negative changes is presented in Table 2. Increasing and decreasing scores did not differ in the ratio of 'within one domain' change or 'between multiple domains' changes.

**Table 2:** Distribution of changes in TFC scores between years

Negative changes	– 1	– 2	– 3	– 4	– 5	– 6	– 7	– 8	– 9	– 10	– 11	– 12	– 13
*N*	1180	496	279	116	82	36	21	9	2	2	1	0	0
Percentage	32.4	16.6	7.7	3.2	2.3	1.0	0.6	0.3	0.1	0.1	0.0	0	0
Cumulative	32.4	49.1	56.7	59.9	62.2	63.2	63.7	64.0	64.0	64.1	64.1	NA	NA

Positive changes	1	2	3	4	5	6	7	8	9	10	11	12	13
*N*	383	135	56	25	23	16	10	4	5	1	2	1	0
Percentage	10.5	3.7	1.5	0.7	0.6	0.4	0.3	0.1	0.1	0.0	0.0	0.0	0
Cumulative	10.5	14.2	15.8	16.5	17.1	17.5	17.8	17.9	18.1	18.1	18.1	18.2	NA

### Changes across TFC domain scores

The most reported first decline in functional capacity, with 61.1%, was in the occupational domain. Furthermore, we saw a percentual shift in the domains across disease stages where the changes occurred; predominantly in occupation in stage I, activities of daily living in stage II, and care level in stages III and IV (Table 3). Comparing premanifest to manifest participants showed a generally consistent pattern of change; however, the manifest group showed a higher percentage of change across all disease stage compared to premanifest participants, this is particularly evident in the care level domain (Table 6).

**Table 3:**
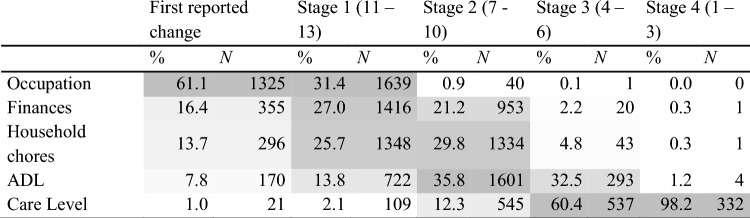
Percentage of change in TFC domain per disease stage and first reported

**Fig. 2:**
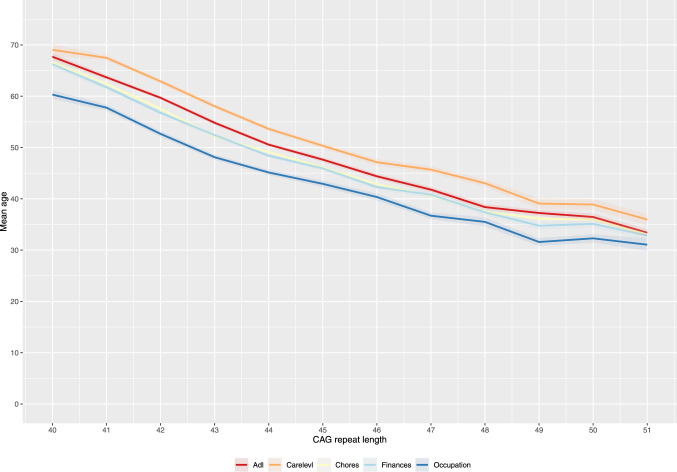
Mean age and standard error per CAG repeat length per TFC domain

Changes across the different domains follow a clear chronological order. A change in occupation is, overall, followed by a change in the ability to perform household chores and finances; this is followed by changes in activities of daily living and care level. This pattern re-emerges, focusing on the mean age at change per domain per CAG repeat (Fig. 2

Overall, the age at change onset per domain differs significantly between all domains, except for the ability to manage finances and perform household chores; *t* (10,450) = 0.410, *p* = 0.994. In Fig. 2, all lines showed a parallel decline and were significantly spaced apart, except for the lines representing finances and household chores, which were overlapping. However, we find a different result when we compare the data grouped according to residential location. A difference in age at change was found regarding the mean age at onset for household chores and finances; *t* (10,450) = 2.750, *p* < 0.050 in Northern America, people lose the ability to manage their finances earlier than those in Europe. Such differences are not found when we group on the basis of sex or education. The results show the same chronological pattern when we correct for predicted age at onset. However, lower levels of occupation are associated with lower predicted age at onset (*β* = 0.254, *p* = 0.0115).

### Exploratory reduced penetrance analysis

**Fig. 3:**
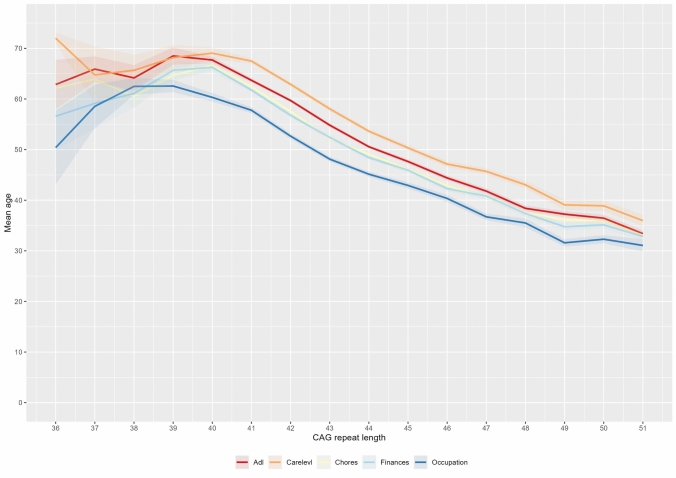
Mean age and standard error per CAG repeat length per TFC domain, including reduced penetrance

The sample size of the RP group (CAG ≥ 36 and ≤ 39) is much smaller (*n* = 113) than that of the FP group (*n* = 1,67). Strikingly, the RP group exhibited a non-linear trend in contrast to the linear pattern observed in the FP group (Fig. 3 in the appendix). The analysis showed that in the group with a CAG-repeat length of 36, the change in occupation occurred earlier with a much greater variance (mean age = 50.4, SE =  ± 7.3 compared to the group with a CAG-repeat length of 40 (mean age = 60.3, SE =  ± 0.9. These results were consistent across all domains. For a detailed overview, refer to Table 5 in the appendix.

## Discussion

The current study shows the general natural course of TFC scores over the years in today's most extensive HD cohort study. One of the primary strengths of the TFC is its simplicity and ease of use, which makes it suitable for routine clinical assessments. The TFC is a well-established scale currently used continuously, although, at best, it is an ordinal scale. The TFC provides us with observational clinical information on functional capacity in HD, especially on the mean and median ages of change over the different domains of functioning. For example, if we focus on those who experienced a change in TFC-score, in the majority of cases (61%), occupational changes are the earliest noted functional change in HD. Additionally, our study found that maintaining occupational status is associated with a later predicted age at onset, emphasizing its importance as a key functional change. These findings are comparable to the 65% reported in a previous study on a much smaller sample [19]. Furthermore, the current study shows that these median and mean ages of functional changes follow a strong linear and noticeable ordinal pattern over various full penetrance HD CAG repeat lengths, affirming the findings originally proposed by Shoulson and Fahn [5]. Moreover, the mean ages at change per domain in the full-penetrance groups are not affected by sex and educational level differences. This consistency reinforces the reliability of the TFC as a measure of functional decline. These findings are consistent with a previous study conducted by Marder et al., which focused on the rate of functional decline. Here, the TFC score was also unaffected by differences in sex or level of education, among other variables (i.e., weight and age at onset) [10].

The current study also examined whether the age at functional change is affected by the continent of residence. We show a striking difference between the ability of individuals in Northern America and those in Europe to manage finances. Northern American participants lose this ability earlier, indicating differences in the manageability between the two regions; the US financial system might be more complex. Furthermore, it is also interesting that in Europe (overall), the mean age at a change in managing finances is similar to the mean age at a change in the ability to perform household chores. Prior research has shown that the rate of functional decline, measured by the TFC in the first two stages of the disease, is almost twice as rapid as in later stages [10]. Future research is warranted as it has not been established, whether these abilities reflect one component of functionality that exacerbates the decline. In that given case, the two domains, the ability to manage finances and do household chores, should be combined to prevent overestimating this particular phase of the disease.

The total functioning capacity assessment is a firmly established assessment widely used in clinical trials as a disease progression measure of HD. For years, the assessment has been recorded in large observational cohorts, two of which are used in the current study. Originally, functionality in HD was represented per category, pinpointing domains in which patients might experience functional decline [5]. Today, it is used as a variable in which a maximum score of thirteen represents normal functioning capacity, and zero an inability to function; a continuous scale. Accordingly, a loss of points represents the progression of the disease. It is also important to note that the TFC represents a loss of capacity, not of activity. Following this, the TFC should not reflect a temporary loss of work, but a loss of capacity to work. Notwithstanding, eighteen per cent of all changes show an improvement in TFC scores, a percentage improvement which is unexpected in a neurodegenerative disease. As mentioned earlier, this improvement might be caused by successful symptomatic interventions, by causes unrelated to HD, or by different researchers assessing the same participant; therefore, some natural interrater differences are expected. Nevertheless, these improvements are found despite standardised training materials. Nowadays, some study protocols aim to minimise interrater variability, for example, by requiring study sites to have the same researcher score the same measures over time. If the accuracy of the TFC assessment improves and the variance and error decrease, the sample required for interventional trials will be reduced.

Focusing further on the methodological aspects of the assessment, the moment we accept that the TFC's scale is continuous, one must accept that, for example, a decline of two points from the maximum score is the same as two steps down from a score of five. In the first case, loss of work function characterises a decline in stage I. In the latter case, the participant loses the ability to complete activities of daily living and possibly has a change in care level (i.e., stage III to IV). Functionally, clinically and in terms of quality of life, the change in the direction of institutionalisation is not equal to losing the ability to work at normal capacity. This is not to say that the TFC should only be used categorically, especially since the last two analyses in this study have shown that the individual functional domains follow a solid ordinal pattern, regardless of CAG-repeat length. The fact that most subjects score 13 and remain fully functional for years suggests a ceiling effect in the TFC. To address this, alternative measures like the FuRST 2.0 scale have been developed to detect earlier functional changes, especially in individuals with subtle impairments not yet captured by the TFC [20]. This scale could complement the TFC in clinical assessments by identifying early declines that the TFC might overlook. We should, however, be aware of these methodological challenges and weaknesses when applying the TFC, especially in clinical trials and individual care.

A key limitation of this study is the low prognostic value of the models for individual patients, which can be attributed to high variability in outcomes. This means that while the models may provide general insights, they are less reliable for predicting specific changes in individual patients. Furthermore, we primarily focused on observational analysis rather than employing extensive statistical analyses. While this approach captures the real-world experiences clinicians encounter, it may overlook important contributing factors influencing functional changes, potentially leading to misattributions of these changes. We accounted for fundamental variables such as sex, education, region of residence, and predicted age at onset—common factors easily determined or readily accessible to healthcare professionals. Interestingly, none of these factors significantly impacted functional change, except for predicted age at onset, where we observed that changes in occupational status may indicate proximity to the age at onset. A future study should focus on the group of people showing increased TFC scores after an initial decline to determine which factors contributed most to this increase in score. In addition to the above, caution should be exercised when utilizing the Total Functional Capacity (TFC) as an outcome measure for individuals with reduced penetrance (RP). Our findings, indicating a deviation from the anticipated linear relationship across Full Penetrance (FP) CAG-repeat lengths, may be influenced by selection bias. One plausible explanation is that many RP individuals in these observational studies have already lost functional capacity due to Huntington's Disease (HD) or other reasons, prompting them to seek medical care. Whereas RP individuals who do not experience these problems, do not seek medical care and do not end up in these cohort studies. Interestingly, in our study participants with RP, who typically experience functional decline later than those with Full Penetrance HD[21], unexpectedly demonstrated earlier occupational challenges at the age of 50 than participants with a CAG of 40. This encourages further research into the use of TFC in this group and highlights the need for future research to unravel the complexities surrounding RP and its impact on clinical measures.

## Conclusion

This study aimed to unveil the natural course of the TFC. Although we have identified methodological limitations in the current use of the TFC, it is a well-established and overall robust functionality measure that describes the progression of ordinal functional decline. We do, however, discourage using the TFC for making far-reaching decisions for participants with reduced penetrance. Also, it is essential that researchers and clinicians bear in mind that an observed increase on the TFC scale may be due to natural variation rather than an actual improvement of functional capacity, attributable to an investigational drug for example.

## Figures and Tables

**Table 6:**
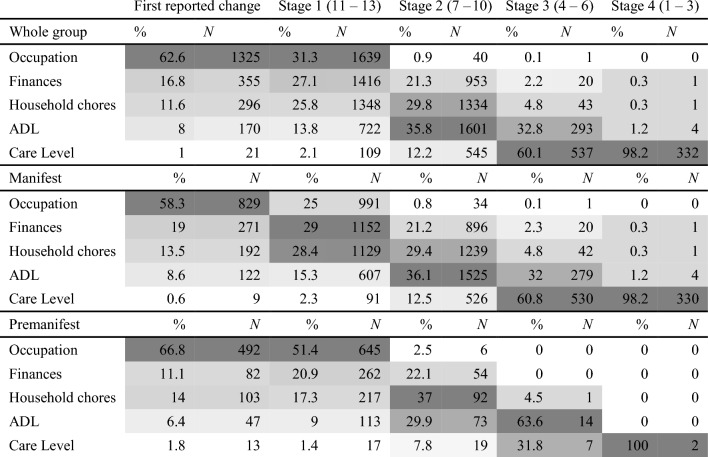
Percentage of change in TFC domain per disease stage and first reported

## Appendix

See Fig. 3, Tables 4, 5 and 6.

**Table 4:** The Total Functioning Capacity Scale.^5^

Occupation
0 = unable
1 = marginal work only
2 = reduced capacity for usual job
3 = normal
Finances
0 = unable
1 = major assistance
2 = slight assistance
3 = normal
Domestic Chores
0 = unable
1 = impaired
2 = normal
ADL
0 = total care
1 = gross tasks only
2 = minimal impairment
3 = normal
Care Level
0 = full time skilled nursing
1 = home or chronic care
2 = home

**Table 5:** CAG-repeat lengths and the age at functional change onset per TFC domain

CAG repeat length	Occupation			Finances			Household Chores			ADL			Care Level		
	Mean	*n*	SE	Mean	*n*	SE	Mean	*n*	SE	Mean	*n*	SE	Mean	*n*	SE
36	50.4	5	7.28	56.6	5	6.71	62.1	7	5.13	62.9	7	4.86	72.0	3	1.15
37	58.5	11	4.28	59.1	8	4.94	63.9	10	3.37	65.9	11	2.52	64.8	4	5.65
38	62.5	31	1.66	61.1	20	2.77	60.5	15	2.23	64.2	19	2.54	65.7	9	3.06
39	62.6	66	1.22	65.7	46	1.86	64.7	54	1.84	68.5	50	1.70	68.2	23	2.46
40	60.3	171	0.87	66.3	188	0.80	66.6	224	0.65	67.7	201	0.65	69.0	102	1.16
41	57.8	243	0.59	61.8	352	0.47	62.4	362	0.42	63.7	333	0.44	67.5	189	0.50
42	52.7	302	0.54	56.8	418	0.42	57.6	486	0.39	59.7	494	0.35	62.9	274	0.45
43	48.1	260	0.49	52.4	353	0.43	52.1	408	0.39	54.8	387	0.40	58.0	231	0.46
44	45.1	190	0.50	48.4	276	0.41	49.1	347	0.34	50.6	323	0.34	53.6	175	0.45
45	42.9	128	0.63	45.9	230	0.45	46.1	274	0.40	47.6	242	0.44	50.3	152	0.53
46	40.3	89	0.71	42.3	152	0.52	42.9	165	0.53	44.4	170	0.50	47.1	113	0.61
47	36.7	52	0.66	40.8	113	0.59	40.6	125	0.50	41.8	134	0.53	45.7	84	0.62
48	35.5	52	0.89	37.4	89	0.59	37.5	85	0.56	38.4	88	0.58	43.0	70	0.71
49	31.6	36	0.76	34.8	63	0.70	36.1	72	0.60	37.2	67	0.59	39.1	39	1.07
50	32.3	27	0.81	35.1	48	0.72	36.0	53	0.65	36.5	42	0.66	38.9	27	1.04
51	31.1	17	1.22	32.8	30	0.85	33.1	42	0.78	33.4	53	0.67	36.0	28	1.13
